# Green synthesis of zinc oxide nanostructures and investigation of their photocatalytic and bactericidal applications†

**DOI:** 10.1039/c9ra07630a

**Published:** 2019-11-18

**Authors:** Mebrahtu Hagos Kahsay, Aschalew Tadesse, Dharamasoth RamaDevi, Neway Belachew, K. Basavaiah

**Affiliations:** Department of Chemistry, Woldia University P.O.BOX 400 Woldia Ethiopia hagosmebrahtu@gmail.com; Department of Applied Chemistry, Adama Science and Technology University Adama P.O.BOX 1888 Ethiopia; AU College of Pharmaceutical Sciences, Andhra University Visakhapatnam 530003 India; Department of Chemistry, Debre Berhan University Debre Berhan P.O.BOX 445 Ethiopia; Department of Inorganic & Analytical Chemistry, Andhra University Visakhapatnam 530003 India

## Abstract

We report a facile one-pot green synthesis of zinc oxide (ZnO) nanostructures using aqueous leaf extract of *Dolichos Lablab* L. as the reducing and capping agent. The optical properties, structure and morphology of the as-synthesized ZnO nanostructures have been characterized by UV-Visible spectroscopy (UV-Vis), Fourier transform infrared spectroscopy (FT-IR), X-ray diffraction (XRD), field emission scanning electron microscopy (FE-SEM) supported with energy dispersive X-ray spectroscopy (EDX), and transmission electron microscopy (TEM). TEM analysis revealed that the as-synthesized ZnO nanostructures have an average particle diameter of 29 nm. XRD patterns confirmed the formation of phase-pure ZnO nanostructures with a hexagonal wurtzite structure. The synthesized ZnO nanostructures were used as a catalyst in the photodegradation of methylene blue (MB), rhodamine B (RhB) and orange II (OII) under visible and near-UV irradiation. The results showed the highest efficiency of photodegradation of ZnO nanostructures for MB (80%), RhB (95%) and OII (66%) at pH values of 11, 9 and 5, respectively, in a 210 min time interval. In addition, the antimicrobial activity of the ZnO nanostructures using the agar well diffusion method against *Bacillus pumilus* and *Sphingomonas paucimobilis* showed the highest zones of inhibition of 18 mm and 20 mm, respectively. Hence, ZnO nanostructures have the potential to be used as a photocatalyst and bactericidal component.

## Abstract 2

We report a facile one-pot green synthesis of zinc oxide (ZnO) nanostructures using aqueous leaf extract of *Dolichos Lablab* L. as the reducing and capping agent.

## Graphical abstract

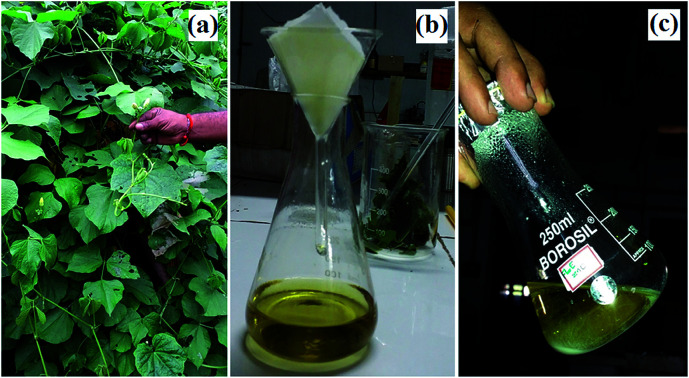

## Introduction

The textile, paper, cosmetic, rubber, food and leather industries dispose of a lot of solid waste and discharge wastewater contaminated with dyes directly or indirectly into rivers, lakes and oceans.^1^ Studies have shown that the dye industry discharges about 100 tons of dyes per year into the environment, such as rivers and water springs.^2,3^ Dye pollution in water bodies can become incorporated into the food chain, leading to the destruction of aquatic ecosystems and potentially resulting in various negative health effects, such as carcinogenic, teratogenic and mutagenic effects.^4,5^ A xanthene class dye, rhodamine B is a cationic dye that is highly soluble in water and is often used as a colorant in textiles and foodstuffs. In addition, it is a well-known fluorescent water tracer.^6^ If it is consumed by any means by humans or animals, it can cause irritation to the skin, eyes and respiratory tract.^7^ Crystal violet is also a cationic dye that has mutagenic and mitotic properties. Dyes such as crystal violet and methylene blue are used in huge quantities in textile and paper dyeing, and worldwide, about 15% of such dyes are released into the environment after use as wastewater. These dye compounds dissolve in water bodies in the concentration range of 10 to 200 mg l^−1^, resulting in serious water pollution globally.^8^ Due to the increasing pollution of water resources, today more than 1.1 billion people lack access to safe drinking water, especially in developing countries. Therefore, it has been a great ambition of researchers to develop new processes and technologies capable of selectively separating and removing target inorganic and organic pollutants from wastewater. Advances in science and technology have resulted in various approaches, such as coagulation, filtration with coagulation, precipitation, ozonation, adsorption, ion exchange, reverse osmosis and advanced oxidation processes, to remove organic pollutants.^9^ Very recently, nano-enabled technologies have shown promising potential in the field of water purification and wastewater treatment.^10^

ZnO nanoparticles (NPs) have a range of applications in new light-emitting devices, solar cells, biosensors, and photocatalysts.^11,12^ Moreover, ZnO NPs are considered to be an effective futuristic water purification material. ZnO NPs also show an extraordinary antibacterial property due to their expanded specific surface area, as the reduced particle size leads to enhanced particle surface reactivity. The bio-safe material, ZnO, can exhibit photo-oxidizing and photocatalysis impacts on chemical and biological species.^12^ In comparison to ZnO NPs synthesized by chemical means, green-synthesized ZnO NPs show vigorous antibacterial effects at a very low concentration.^13,14^ ZnO NPs have diverse applications in fields such as anticancer, antidiabetic, antibacterial and antifungal treatments, drug delivery, and agricultural technologies.^15–19^ Their nanosized nature leads to changes in the chemical, mechanical, electrical, structural, morphological and optical properties of the material, which enables NPs to interact in a unique manner with cell biomolecules, facilitating the physical exchange of NPs into inner cellular structures.^20^

Even though the synthesis of ZnO NPs *via* green synthesis routes using biological systems such as bacteria, fungi, yeast, and plants^21,22^ has been reported, it has been mentioned that handling and controlling plant material is easier when compared to the other biological systems. ZnO NPs have been prepared *via* plant-mediated synthesis using various plant species.^23–33^ Extracts of these plants have been employed as reducing and stabilizing agents during the green synthesis of various sized ZnO nanostructures. To the best of our knowledge, *Dolichos lablab* Linn has never been reported for the synthesis of ZnO nanostructures. Thus, we decided to use this plant species due to its wide availability, low cost, edible nature, low toxicity and solubility in water as an eco-friendly solvent. *Dolichos lablab* L. belongs to the family Leguminosae (Fabaceae). The genus *Lablab* is native to India^34^ and is widespread in every corner of the world. *Dolichos lablab* L. is rich in minerals, vitamins, proteins, essential amino acids, dietary fiber, starch, flavonoids, steroids, glycosides, trypsin inhibitors, hydrogen cyanide, oxalate, haemagglutinin units, phytate, tannin, saponin, alkaloids and polyphenol.^35,36^

In this paper, ZnO nanostructures were synthesized using a facile green synthesis approach, with *Dolichos lablab* L. leaf extract used as a reducing and capping agent. The as-prepared ZnO nanostructures were characterized using different spectroscopy and microscopy instruments. The photocatalytic efficiency of the ZnO nanostructures was studied under combined visible and near-UV photoirradiation for three model organic dye pollutants, *i.e.*, MB, RhB, and OII. Furthermore, the antimicrobial activity of the green-synthesized ZnO nanostructures was evaluated against pathogenic Gram-positive (*Bacillus pumilus*) and Gram-negative (*Sphingomonas paucimobilis*) microorganisms.

## Experimental

### Materials

All chemicals used in this study were analytical grade and were used without further purification. Zinc acetate dihydrate extra pure (Zn(CH_3_COO)_2_·2H_2_O) and purified sodium hydroxide pellets (NaOH) were obtained from Merck, India. MB, RhB and OII were received from Sigma-Aldrich. The metal halide lamp (visible (452.5 W m^−2^) and UV (70.2 W m^−2^) photoirradiation intensity) was from Fast track, India. The leaves of *Dolichos lablab* L. were collected from Andhra University, Visakhapatnam, India. The plant was authenticated by Dr S. B. Padal, voucher specimen number – AU (AUH) 22232, in Andhra University Herbarium, Botany Department, Andhra University, Andhra Pradesh, India. Sources of standard strains of Gram-positive (*Bacillus pumilus*) and Gram-negative (*Sphingomonas paucimobilis*) bacteria were from Adhya Biosciences Pvt. Ltd., Visakhapatnam.

### Preparation of aqueous leaf extract of *Dolichos lablab* L.

**Fig. 1:**
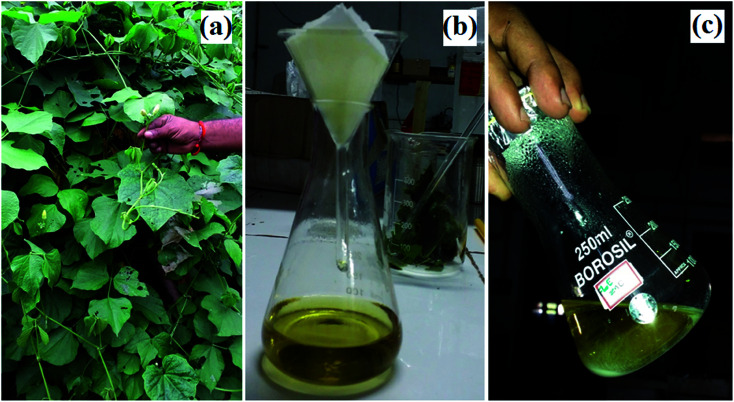
Images of *Dolichos lablab* L.: (a) habitat, (b) leaf extract, and (c) ZnO nanostructures.

*Dolichos lablab* L. leaves were collected and then washed with double distilled water to remove any dust. 20.0 g of leaves of *Dolichos lablab* L. (Fig. 1a) were weighed and heated in 100 ml of Milli-Q water at 70 °C for 30 min. The resultant extract was allowed to cool and then filtered with Whatman no. 42 filter paper to produce greenish yellow filtrate (Fig. 1b). Finally, the obtained aqueous extract was stored in a refrigerator at 4 °C for the synthesis of ZnO nanostructures.

### Synthesis of zinc oxide nanostructures using aqueous leaf extract of *Dolichos lablab* L.

In a typical synthesis, 10.0 ml (1%) of aqueous leaf extract of *Dolichos Lablab* L., 0.5 g (2.73 mmol) zinc acetate dihydrate, and 10.0 mmol NaOH and 80.0 ml of Milli-Q water were refluxed at 70 °C under magnetic stirring for 1 h until a pale white precipitate formed, which indicated the synthesis of ZnO nanostructures (Fig. 1c). The formed ZnO nanostructures were centrifuged and washed periodically with Milli-Q water and ethanol in order to remove unreacted precursors. Finally, ZnO precipitate was dried under vacuum at room temperature.

### Characterization of zinc oxide nanostructures

Optical properties were analyzed using UV-Vis spectrophotometers (UV-2600 SHIMADZU) and (UV-500, Thermo Electron Corporation) in the wavelength range of 200–800 nm. FT-IR spectra were recorded over the range of 4000–400 cm^−1^ using a SHIMADZU-IR PRESTIGE-2 spectrometer. Powder XRD patterns were recorded on a PANalytical X'pert Pro diffractometer at 0.02 degree per second scan rate using CuKα_1_ radiation (*λ* = 1.5406 Å). The morphology and elemental composition were characterized using FE-SEM (JEOL, JSM-7600F) at an accelerating voltage of 0.1 to 30 kV, equipped with EDX. The size and shape of the ZnO nanostructures were investigated by TEM (TEM model FEI TECNAI G2 S-Twin).

### Photocatalytic activity of ZnO nanostructures

The photocatalytic degradation of three organic dye pollutants, *i.e.* MB, RhB and OII, using the synthesized ZnO nanostructures as a semiconductor catalyst system in a batch reactor under visible and near-UV photoirradiation, was investigated. Different initial concentrations of dye solution (5, 25, 50 and 100 ppm) were prepared from dye powder, with the pH value of the solution maintained at pH = 5, 9, 11, and poured into a 100 ml beaker. 5 ppm model organic dye, 1 g l^−1^ ZnO nanostructure powder and 10 μl 30% H_2_O_2_ (to increase the concentration of dissolved oxygen) were mixed to form a suspended dye solution, and the beaker was covered with aluminum foil to keep the system dark for the first 30 min under magnetic stirring. At the adsorption–desorption equilibrium, 3 ml of the colloidal suspension was taken into a clean vial and labeled as 0 min. The subsequent batches were irradiated with a metal halide lamp (visible and near-UV) while being magnetically stirred to ensure homogeneous mixing. Similarly, at every 30 min time interval, 3 ml of the colloidal suspension was removed and placed into a vial, and labelled as 30, 60, 90, 120, 150, 180 and 210 min. Finally, the absorption of each solution was measured in the wavelength range from 200 to 800 nm using UV-Vis spectroscopy and the percentage of dye degradation (%) was calculated using eqn (1).

1 Dye degradation (%) = (*C*_0_ − *C*)/*C*_0_ × 100

*C*_0_ is the initial concentration of dye (ppm) before the addition of catalyst and photoirradiation and *C* is the concentration of dye at equilibrium (ppm) after the addition of catalyst and exposure to photoirradiation.

### Antibacterial activity screening

The antimicrobial activity was assessed by employing 24 h cultures with ZnO nanostructures by using the agar well diffusion method.^37^ The medium was sterilized by autoclaving at 120 °C (15 lb in^−2^). About 20 ml of nutrient agar medium/potato dextrose agar seeded with the respective strains of bacteria was transferred aseptically into each sterilized Petri plate. The plates were left at room temperature for solidification. In each plate, a single well of 6 mm diameter was made using a sterile borer. The ZnO nanostructures were freshly reconstituted with suitable solvent (DMSO) and tested at various concentrations (2.5, 5, and 10 mg ml^−1^). The samples, control, and standard (Ciprofloxacin) were placed in 6 mm diameter wells. 5 μg ml^−1^ standard was used as a positive control. Assays were incubated at 37 ± 2 °C. The activity diameter of the zone of inhibition was measured using the Himedia antibiotic zone scale.

## Results and discussion

The green synthesis of ZnO nanostructures using leaf extract of *Dolichos lablab* L. provides a simple, low cost and environmentally friendly route without the use of toxic organic solvents and hazardous materials. Moreover, in this procedure, there is no need to use high temperature, pressure or energy. Aqueous leaf extract of *Dolichos lablab* L. performed as a reducing and capping agent during ZnO nanostructure synthesis without releasing toxic chemicals to the environment. The preliminary phytochemical analysis of the present plant extract is presented in Table 1. The results demonstrated that alkaloid, phenol, flavonoid, amino acid, protein, terpenoid and saponin were the qualitatively identified constituents of *Dolichos lablab* L. in this study, in agreement with previous reports.^38,39^

**Table 1:** Preliminary phytochemical investigation of aqueous leaf extract of *Dolichos lablab* L.^a^

S. no.	Test	Chemical constituents	Result
1	Mayer's test	Alkaloid	+
2	FeCl_3_ solution	Phenol	+
3	NaOH	Flavonoid	+
4	Fehling solution	Carbohydrate	−
5	Conc. HNO_3_	Amino acid and protein	+
6	Methanol, chloroform, H_2_SO_4_	Terpenoid	+
7	Shake with water	Saponin	+

a Where, (+) indicates presence and (−) indicates absence.

### UV-Vis analysis

**Fig. 2:**
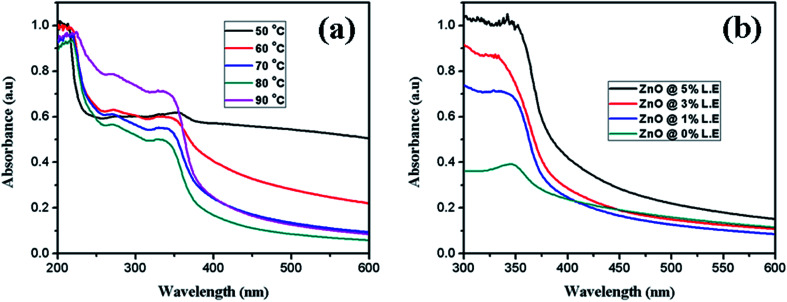
UV-Vis absorption spectra of synthesized ZnO nanostructures at different (a) temperatures, (b) percentages of leaf extract.

The absorption spectrum of synthesized ZnO nanostructures using aqueous leaf extract of *Dolichos Lablab* L. is presented in Fig. 2. Synthesis of ZnO nanostructures was optimized by increasing the temperature of the reaction from 60 °C to 90 °C (Fig. 2a) and volume of the plant extract from 0% to 5% (Fig. 2b). The characteristic absorption peak was observed from 330 to 354 nm at different temperatures. The results indicate that the intensity of the absorption peak of the synthesized ZnO nanostructures increased with increasing temperature of the reaction and percentage of plant extract. When the reaction mixture was optimized to 90 °C, a characteristic peak was observed at 329 nm. Mang *et al.*^40^ reported a similar result. However, ZnO nanostructures synthesized using 5% plant extract showed a characteristic peak at 342 nm. In addition, increasing the temperature of the reaction mixture and concentration of plant extract showed a blue shift, *i.e.*, a decrease in the size of the ZnO nanostructures.

### UV-DRS and FT-IR analysis

**Fig. 3:**
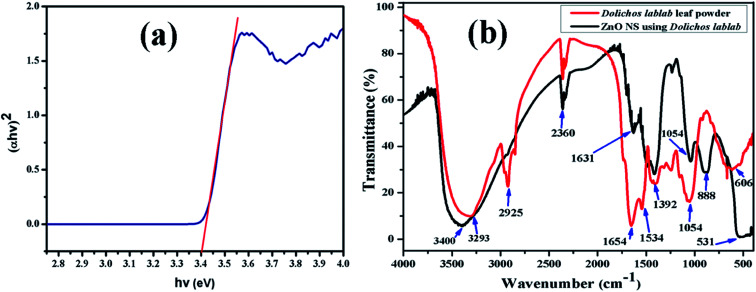
(a) Tauc plot of ZnO nanostructures, (b) FT-IR spectra of aqueous leaf extract of *Dolichos lablab* L. and the as-synthesized ZnO nanostructures.

The ZnO nanostructure surface absorbed radiation in the UV region at *λ* = 366 nm, and its band gap was extrapolated to be 3.4 eV from the Tauc relation (eqn. (2)). In the literature, the band gap of ZnO NPs synthesized using *Abutilon indicum* plant extract is reported to be 3.37 eV at a wavelength of 368 nm.^40,41^Fig. 3a represents the Tauc plot of the as-synthesized ZnO nanostructures. The photocatalytic efficiency of the ZnO nanostructures under visible and near-UV photoirradiation is limited due to its wide band gap. In fact, the band gap of ZnO confines its photocatalytic activity within the UV light range. As a result, it can utilize only 4% of the incident solar radiation.^42^ Therefore, a very small amount, *i.e.*, 10 μl 30% H_2_O_2_, was added to the dye solution to increase the concentration of dissolved oxygen during degradation.

2 *αhν* = *A*′(*hν* − *E*_g_)^*n*^

Here, *α* is the absorption coefficient (*α* = 2.303*A*/*t*), *A* is absorbance, *t* is the path length of the wave equal to the thickness of the cuvette, *A* is a proportionality constant, *hν* is the photon energy and *E*_g_ is the energy band gap for the allowed direct transition of value *n* equal to 1/2.

FT-IR analysis was done to identify the possible functional groups involved in the reduction of zinc ions and the capping of reduced ZnO nanostructures. The FT-IR spectrum of the leaf extract of *Dolichos lablab* L. is shown in Fig. 3b, which shows absorption bands at 3293, 2925, 2360, 1654, 1534, 1392, 1054 and 606 cm^−1^, due to hydrogen bonded O–H stretching of alcohol or phenol functional groups, sp^3^ C–H stretching of alkane groups, CO_2_ interference, C=O stretching of amide groups, N–O asymmetric stretching, N–O symmetric stretching, C–O stretching of carboxylic and ester groups and C–C bending.^43^ The FT-IR spectrum of *Dolichos lablab* L. mediated ZnO nanostructures shows absorption bands at 3400, 2360, 1631, 1392, 1044, 888 and 531 cm^−1^, due to hydrogen bonded O–H stretching of alcohol or phenolic groups, CO_2_ interference, C=O stretching of amide, N–O symmetric stretching, C–O stretching of carboxylic acid, CH_3_ wag and Zn–O bending vibrations. Hence, phytoconstituents of *Dolichos lablab* L. leaf extract were used as capping and reducing agents. Similarly, Udayabhanu *et al.*^44^ reported the Zn–O bending vibration of ZnO super-structures synthesized using skin extract of *Vitis labrusca* to be 532 cm^−1^.

### XRD analysis

**Fig. 4:**
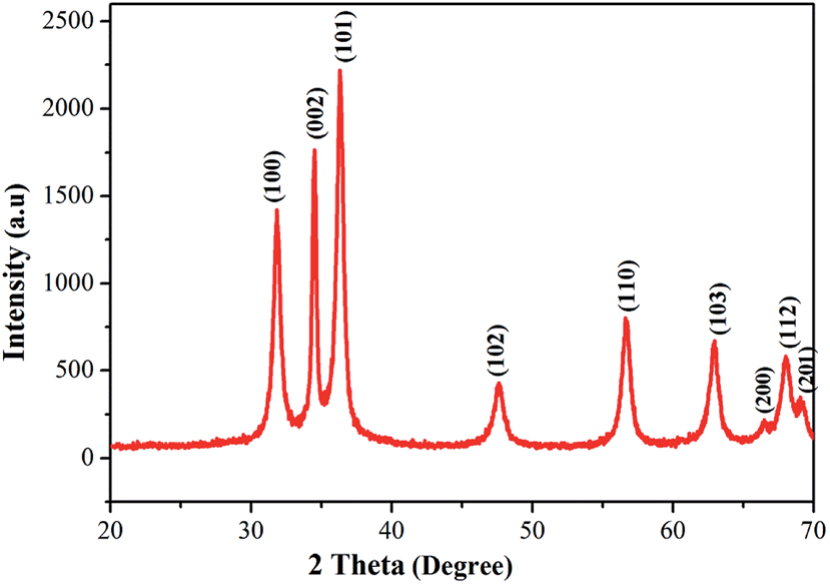
Powder XRD pattern of ZnO nanostructures synthesized using aqueous leaf extract of *Dolichos lablab* L.

The phase purity and crystallinity of the as-prepared ZnO nanostructures was investigated using X-ray diffraction. The XRD patterns of the obtained ZnO nanostructures are shown in Fig. 4. The XRD patterns of the ZnO nanostructures show 2*θ* values at 31.84°, 34.52°, 36.33°, 47.63°, 56.64°, 62.91°, 66.54°, 68.05° and 69.12°, which correspond to (100), (002), (101), (102), (110), (103), (200), (112) and (201) planes of hexagonal wurtzite structure (JCPDS card no.: 36-1451).^45^ The broadening of the diffraction peaks clearly indicates the presence of nanoparticles in the product. In this study, an unusual larger intensity peak, which is deviant when compared to the standard and other studies, is observed at 2*θ* = 34.52° (002). The average crystallite size (*D*) of the synthesized ZnO nanostructures was calculated using the Debye–Scherrer formula (eqn. (3)) to be 36 nm.^46^

3 *D* = 0.9*λ*/*β* cos *θ*

*D* is the crystallite size (nm), *λ* is the wavelength of CuKα radiation (0.15406 Å), *β* is the full width at half maximum of the diffraction peak (in radian). Table 2 illustrates the structure and geometric parameters of the ZnO nanostructures.

**Table 2:** Structure and geometric parameters of ZnO nanostructures

2*θ* (degree)	FWHM (*β*)	*d*-spacing (Å)	cos *θ*	Crystallite size (nm)
8.3932	1.3056	10.52625	0.99732	6.38
31.8365	0.4080	2.80859	0.96165	21.16
34.5150	0.2448	2.59651	0.95498	35.51
36.3340	0.1224	2.47059	0.95015	71.38
47.6293	0.5712	1.90772	0.91486	15.88
56.6423	0.0816	1.62369	0.88030	115.55
62.9124	0.2448	1.47610	0.85304	39.75
66.5366	0.5712	1.40422	0.83611	17.38
68.0506	0.4488	1.37662	0.82879	22.31
69.1192	0.6528	1.35792	0.82354	15.44
Average				36.07

### FE-SEM and EDX analysis

**Fig. 5:**
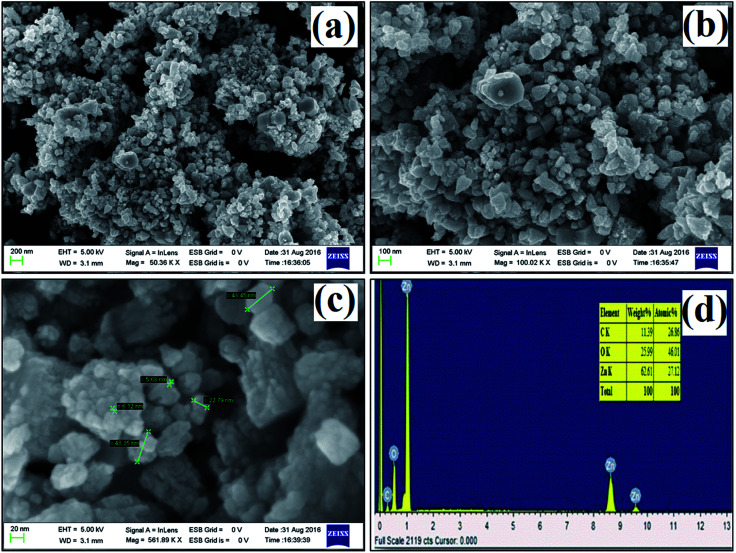
(a–c) FE-SEM images at different magnifications, (d) EDX spectrum of ZnO nanostructures synthesized using aqueous leaf extract of *Dolichos lablab* L.

The size and morphology of the synthesized ZnO nanostructures were studied using FE-SEM. Fig. 5a–c show FE-SEM images of the synthesized ZnO nanostructures under different magnifications, and their diameter range is 7 to 49 nm. Hexagonal and triangular ZnO nanostructures are observed in Fig. 5a and b. The elemental composition of the synthesized ZnO nanostructures was confirmed by EDX, as shown in Fig. 5d. The elemental composition of Zn, O and C in the ZnO nanostructures was found to be 27.1%, 46.0% and 26.9% by atomic mass, respectively. The presence of C in the peak indicates that organic molecules from the plant extract were used as capping agents during the formation of the ZnO nanostructures.

### TEM analysis

**Fig. 6:**
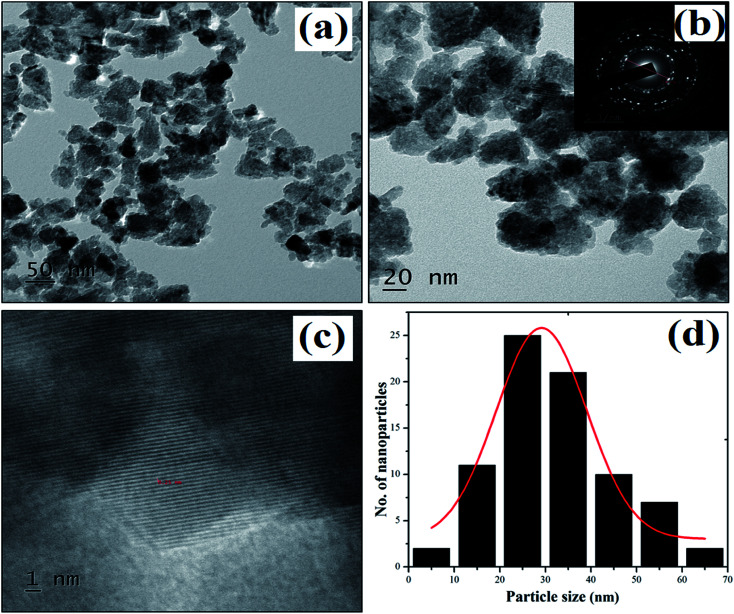
(a–c) TEM images of plant mediated ZnO nanostructures and selected area electron diffraction patterns. (d) Particle size distribution histogram.

The TEM images in Fig. 6 show irregularly shaped ZnO nanostructures. The diffraction rings in the SAED pattern match the XRD crystal planes, *i.e.*, (100), (002), (101), (102), (110), (103), (200), (112) and (201). The average particle size of the as-synthesized ZnO nanostructures was found to be 29 nm. The less intense layering at the surface of the nanoparticles in Fig. 6 proves that the plant extract was also used as a capping agent.^47^

**Fig. 7:**
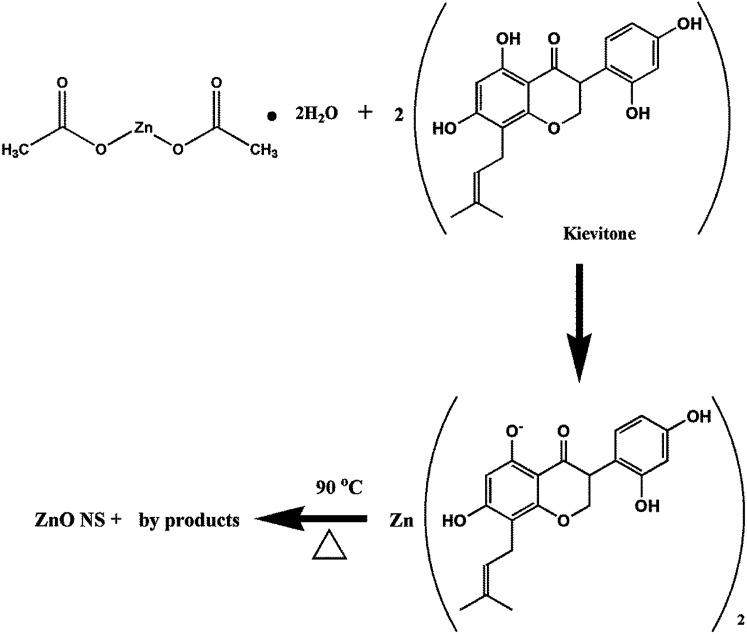
Possible mechanism of synthesis of ZnO nanostructures using bioactive molecules.

The formation of ZnO nanoparticles using zinc acetate in basic medium can be explained *via* different reactions such as dissolution, hydrolysis and precipitation.^48^ The proposed mechanism for the synthesis of the ZnO nanostructures using zinc acetate dihydrate and one of the biologically active phytoconstituents of *Dolichos lablab* L. as a reducing and capping agent is schematically illustrated in Fig. 7.^49^

### Kinetic studies of dye degradation using ZnO nanostructures as photocatalyst

**Fig. 8:**
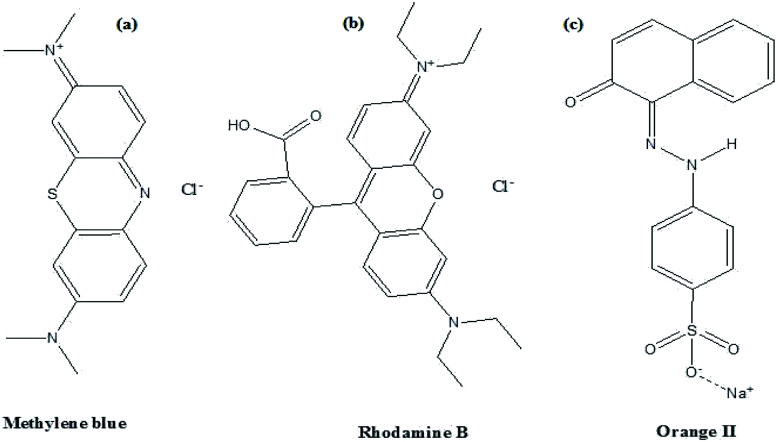
Structures of (a) Methylene blue, (b) rhodamine B and (c) Orange II dyes.

**Fig. 9:**
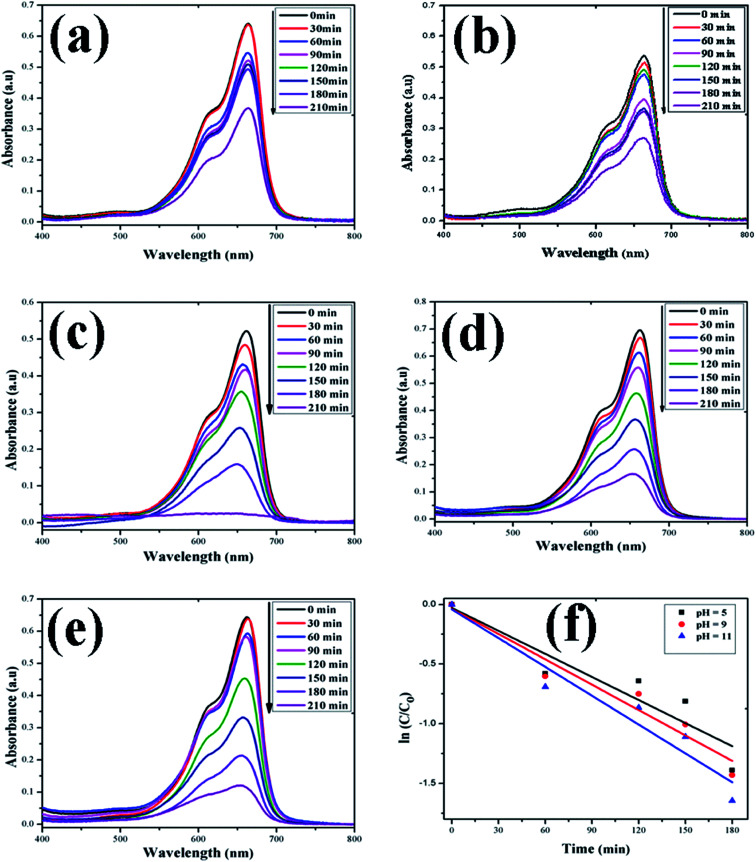
Time-dependent UV-Vis spectra of (a) MB dye without ZnO; (b) MB dye + H_2_O_2_; (c) MB dye + ZnO + H_2_O_2_ at pH = 5; (d) MB dye + ZnO + H_2_O_2_ at pH = 9; (e) MB dye + ZnO + H_2_O_2_ at pH = 11. (f) First-order kinetic model of MB at different pH values.

**Fig. 10:**
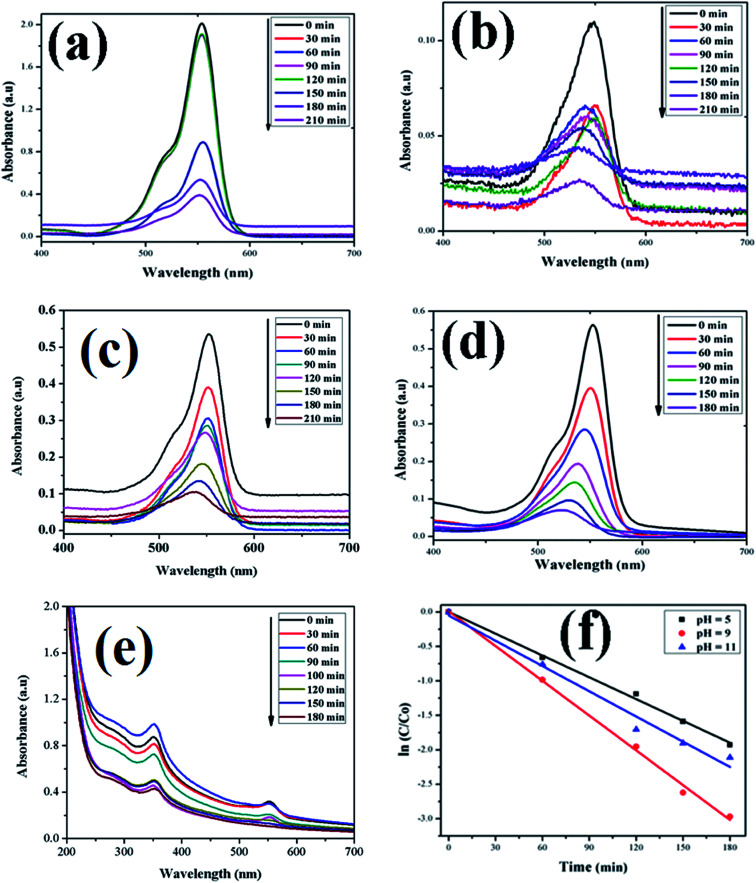
Time-dependent UV-Vis spectra of (a) RhB dye without ZnO; (b) RhB dye + H_2_O_2_; (c) RhB dye + ZnO + H_2_O_2_ at pH = 5; (d) RhB dye + ZnO + H_2_O_2_ at pH = 9; (e) RhB dye + ZnO + H_2_O_2_ at pH = 11. (f) First-order kinetic model of RhB at different pH values.

**Fig. 11:**
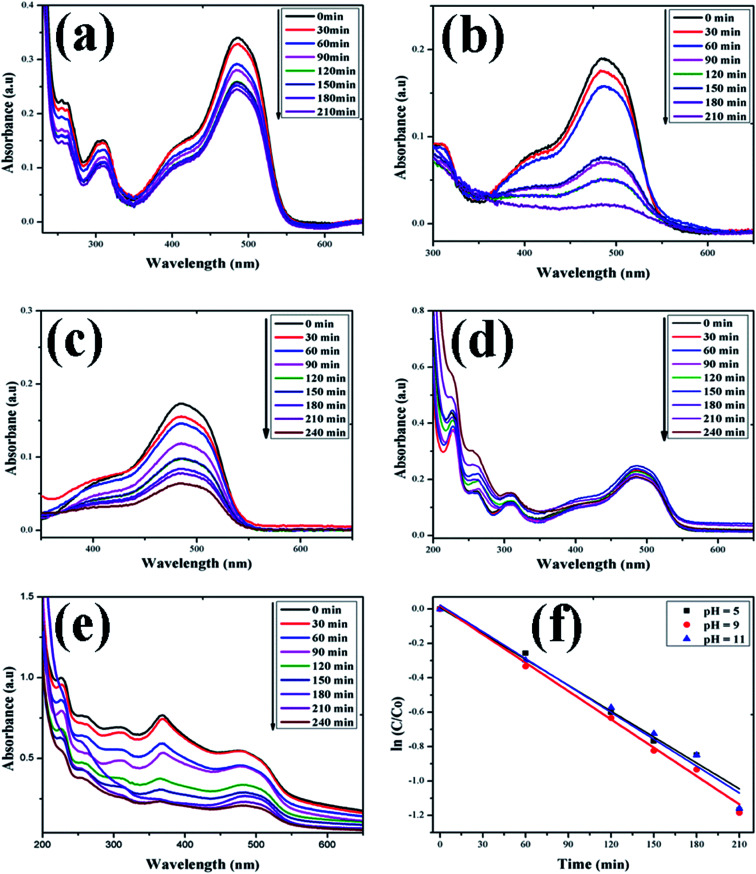
Time-dependent UV-Vis spectra of (a) OII dye without ZnO; (b) OII dye + H_2_O_2_; (c) OII dye + ZnO + H_2_O_2_ at pH = 5; (d) OII dye + ZnO + H_2_O_2_ at pH = 9; (e) OII dye + ZnO + H_2_O_2_ at pH = 11. (f) First-order kinetic model of RhB at different pH values.

In this study, the as-synthesized ZnO nanostructures were used as a catalyst to degrade three organic dye pollutants, *i.e.*, MB, RhB and OII, under visible and near-UV photoirradiation. The maximum absorption of the dye was observed at 664 nm, 555 nm and 487 nm, respectively. Fig. 8 shows the chemical structures of the three organic dyes. 50 ml (5 ppm) organic dye was photodegraded using 1 g l^−1^ of ZnO nanostructures under visible and near-UV photoirradiation, and the photocatalytic degradation efficiency of the ZnO nanostructures was studied at different initial dye concentrations, time intervals and pH values. Fig. 9–11 show time-dependent UV-Vis spectra of organic dyes and first-order kinetic modeling under different pH conditions. The speed of the photocatalytic reaction or decolorization of dye for all three dyes increases with time span. The percentage of dye degradation was calculated using eqn (1).

A kinetic study of the catalytic degradation of the organic dyes using the ZnO nanostructures was performed using the Langmuir–Hinshelwood–Hougen–Watson (LH–HW) kinetic model (eqn. (4)).^50,51^ For a hypothetical reaction, A (dye) → products. The algebraic form of the rate law, *r*_A_ may be a linear function of the concentration, as −*r*_A_ = *kC*_A_, or it may be some other algebraic function of the concentration, as −*r*_A_ = *kC*_A_^2^. However, for the photocatalytic degradation of organic dye that considers the adsorption properties of the reactant on the photocatalyst surface (*e.g.* ZnO), the LH–HW rate law is expressed as:

4 −*r*_A_ = *kC*_A_/(1 + *kC*_A_)

Here, −*r*_A_ is the rate of disappearance of organic dye (mol s^−1^ dm^−3^), *C*_A_ is organic dye concentration (mol dm^−3^), and *k* is the rate constant.

After rearrangement and integrating both sides of eqn (4), a plot of ln(*C*/*C*_0_) *vs. t* under different pH conditions was fitted to the LH-HW kinetic model, as shown in Fig. 9f, 10f and 11f. The reaction rate constants (*k*) for photocatalytic degradation of MB, RhB and OII dyes were 9.14 × 10^−3^ min^−1^, 1.65 × 10^−2^ min^−1^ and 5.10 × 10^−3^ min^−1^, respectively. Similarly, their corresponding *R*^2^ values were 0.829, 0.996 and 0.994, respectively. It can be concluded that the photocatalytic degradation of MB, RhB and OII dyes using the ZnO nanostructure photocatalyst follows a pseudo-first-order kinetic model in this study. However, Rana *et al.*^52^ reported that the photocatalytic degradation of RhB (5 ppm) using ZnO NPs synthesized from *Terminalia chebula* fruit extract had a rate constant of 0.228 h^−1^ and degradation efficiency of 70% after 5 h.

**Fig. 12:**
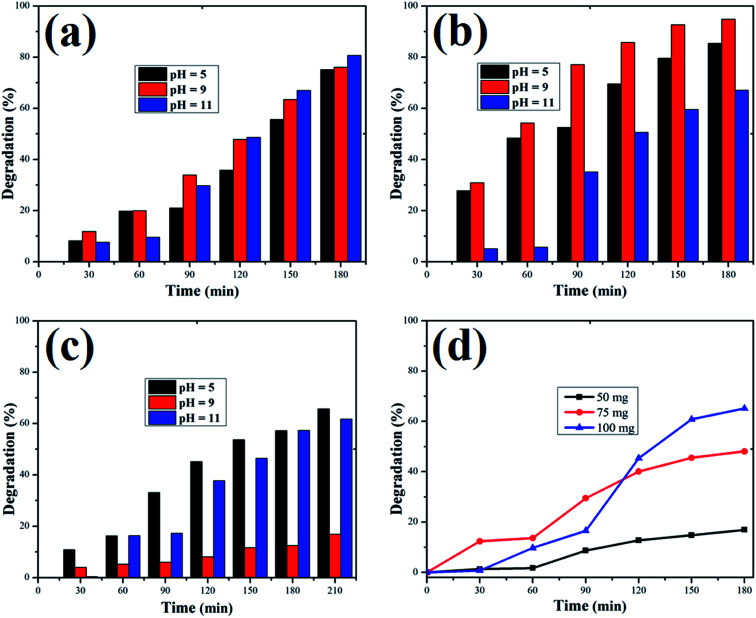
Percentage of dye degradation *versus* time at pH 5, 9 and 11 for (a) Methylene blue, (b) rhodamine B., and (c) Orange II dye solutions. (d) Effect of catalyst dose on Methylene blue degradation.

The adsorption of organic dyes on the surface of ZnO nanostructures was influenced by the pH of the dye solution. Varying the pH of the dye solution changed the value of the percentage of dye degradation for MB, RhB and OII, as shown in Fig. 12a–c. Similarly, the catalyst dose of the ZnO nanostructures also influenced the photocatalytic degradation of MB (Fig. 12d). The photocatalytic degradation of dyes increased with increasing photocatalyst dose per adsorbent (0.5 g l^−1^, 0.75 g l^−1^, 1 g l^−1^), mainly due to an increase in the number of active sites on the photocatalyst surface. Moreover, organic dye degradation was found to be pH dependent and the highest percentage of dye degradation was observed for MB at pH = 11 (80%), RhB at pH = 9 (95%) and OII at pH = 5 (66%). However, in the absence of ZnO nanostructures, the three dyes degraded slowly. Increasing the pH value for the cationic dyes (MB and RhB) increased their photocatalytic degradation, mainly due to the production of more populated hydroxyl ions on the surface of the photocatalyst under visible and near-UV photoirradiation. In contrast, increasing the pH value of the anionic dye (OII) decreased its photocatalytic degradation, due to fewer hydroxyl radical formations on the surface of the photocatalyst under visible and near-UV photoirradiation. Therefore, the pH of the dye solution played a major role in the formation of stable dye–ZnO nanostructure complexes. The optimized pH values for the photodegradation of MB, RhB and OII were 11, 9 and 5, respectively. Increasing the time of contact between the photocatalyst (ZnO nanostructures) and organic dye facilitated photodegradation of all organic dyes under visible and near-UV photoirradiation. The photodegradation of all the three organic dyes followed the decreasing order of: organic dye + ZnO NS + H_2_O_2_ > organic dye + ZnO NS > organic dye alone.

Reports show that green-synthesized ZnO NPs have been used in different applications such as photocatalysis for the removal of organic dye pollutants^41,53–55^ and as a bactericide.^41,54,56–58^ In the present work, the photocatalytic degradation of RhB under near-UV and visible irradiation using the as-synthesized ZnO nanostructures showed 95% degradation at pH = 9, and a similar result has been reported by Nagaraja *et al.*^59^ It is better to use combined UV and visible irradiation rather than only UV light for the catalytic degradation of organic dye pollutants, since UV radiation can be dangerous. *Dolichos lablab*-capped ZnO nanostructures photocatalytically degraded MB, RhB and OII dyes better than commercial ZnO NPs, which degrade MO, MB and RhB dyes by 68.1%, 75.5% and 65.7%, respectively, under UV irradiation.^60^ Under UV irradiation, protein-capped ZnO NPs degrade MB dye at a reaction rate (*k*) of 3.27 × 10^−3^ s^−1^,^19^ while in the present study, MB dye was degraded at a reaction rate of 9.14 × 10^−3^ min^−1^ (1.52 × 10^−4^ s^−1^), indicating that the rate of degradation is faster in the present study.

**Fig. 13:**
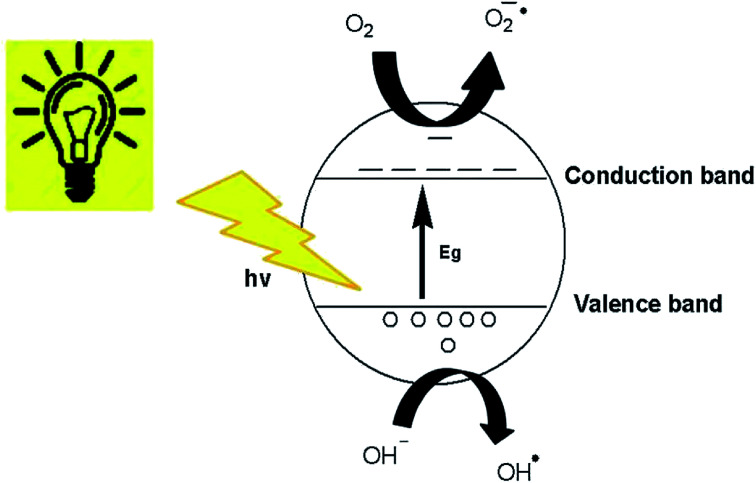
Photocatalytic mechanism of ZnO nanostructures.

Generally, it can be concluded that the large surface area to volume ratio and large number of active sites on the ZnO nanostructures, where the photogenerated charge carriers are able to interact with the adsorbed organic dyes to form hydroxyl or superoxide radicals, facilitated the decolorization of organic dye molecules to CO_2_ and water.^61^ The degradation of dyes in this study occurred due to the photocatalytic process and dye sensitization.^62^ The schematic diagram of the photocatalytic mechanism over the surface of the ZnO nanostructures is presented in Fig. 13.^49,63^ Organic dye degradation is caused by the photogeneration of hole–electron pairs between the valence and conduction bands. Photogenerated h^+^ (hole) in the valence band reacts with either H_2_O or OH^−^ to produce the OH˙ radical.^64^ Aside from OH^−^, various species involved in the degradation mechanism are O_2_˙^−^ and HO_2_˙.^65^h^+^ + H_2_O → OH˙ + H^+^h^+^ + OH^−^ → OH˙

On the other hand, e^−^ in the conduction band reacts with adsorbed O_2_ on the ZnO nanostructure surface to generate O_2_˙^−^ and then OH˙ radicals.^66^e^−^ + O_2_ → O_2_˙^−^2O_2_˙^−^ + H^+^ → HO_2_˙ + O_2_˙^−^2HO_2_˙ → H_2_O_2_ + O_2_H_2_O_2_ + e^−^ → OH˙ + OH^−^H_2_O_2_ + h^+^ → 2OH˙

Organic dye degradation or decolorization in the presence of the most reactive radicals can be generalized as follows.Dye + (O_2_˙^−^ or OH˙ or HO_2_˙) → intermediate + products

### Antibacterial activity of ZnO nanostructures

**Fig. 14:**
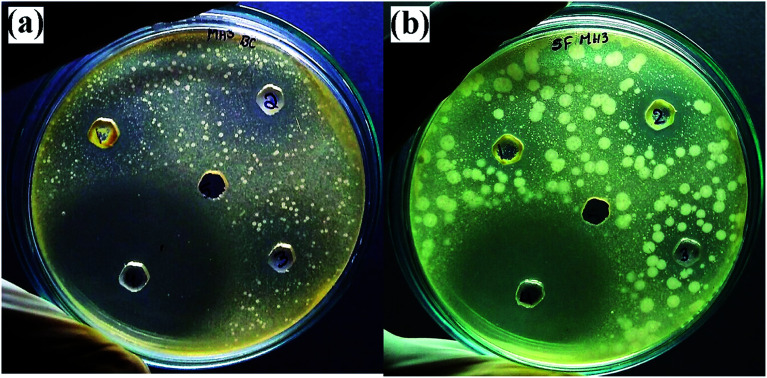
Antimicrobial activities of synthesized ZnO nanostructures against (a) *Bacillus pumilus*, and (b) *Sphingomonas paucimobilis*.

The antimicrobial activity of ZnO nanostructures against standard strains of Gram-positive (*Bacillus pumilus*) and Gram-negative (*Sphingomonas paucimobilis*) bacteria is shown in Fig. 14 and Table 3. A higher zone of inhibition was observed in *Sphingomonas paucimobilis* with a zone of inhibition of 20 mm, whereas the inhibition zone in *Bacillus pumilus* was 18 mm at 5.0 mg ml^−1^ ZnO nanostructures, mainly due to the thick peptidoglycan layers in Gram-positive bacteria. However, in the negative control group (DMSO), no zone of inhibition was observed, whereas a standard (Ciprofloxacin) zone of inhibition of 40 mm was observed for both types of bacteria.

**Table 3:** Zone of inhibition of ZnO nanostructures against pathogens^a^

S. no.	Pathogens	Concentration of ZnO nanostructures (mg ml^−1^)			Standard (Ciprofloxacin)	Control (DMSO)
		2.5	5.0	10.0	5 μg/ml	
1	*Bacillus pumilus*	12 mm	18 mm	15 mm	40 mm	NA
2	*Sphingonomas paucimobilis*	15 mm	20 mm	15 mm	40 mm	NA

a Where, DMSO is dimethyl sulfoxide and NA is not available.

Hexagonal wurtzite structured ZnO NPs biosynthesized using the *Pichia kudriavzevii* yeast strain has previously been reported to show antimicrobial activity against both Gram-positive bacteria and Gram-negative bacteria, in which the zone of inhibition against *B. subtilis* (Gram-positive) was 9 mm.^67^ However, our green-synthesized ZnO nanostructures using *Dolichos lablab* L extract showed a zone of inhibition of 18 mm against *Bacillus pumilus*, which is also Gram-positive. The zone of inhibition of our green-synthesized ZnO nanostructures against *Sphingomonas paucimobilis* showed a better zone of inhibition than that reported by Safawo *et al.*^58^.

## Conclusions

Hexagonal wurtzite and irregularly shaped ZnO nanostructures were synthesized using aqueous leaf extract of *Dolichos lablab* L. *via* a green synthesis approach. The phytoconstituents such as phenolic compounds and proteins present in the *Dolichos lablab* L. were found to be responsible for the formation and capping of ZnO nanostructures. The formation of the ZnO nanostructures was confirmed by UV-Vis, UV-DRS, FT-IR, XRD, FE-SEM supported with EDX and TEM. UV-DRS revealed a band gap of 3.4 eV, indicating the semiconducting behavior of the ZnO nanostructures. Using ZnO nanostructures as a photocatalyst for the degradation of organic dyes, MB, RhB and OII were photodegraded by 80, 95, and 66%, respectively. The antimicrobial activity of the ZnO nanostructures using the agar well diffusion method against *Bacillus pumilus* and *Sphingomonas paucimobilis* showed zones of inhibition of 18 mm and 20 mm, respectively. Hence, the ZnO nanostructures could be used as a semiconductor photocatalyst as well as a bactericide for pathogenic bacteria during wastewater treatment.

## Conflicts of interest

The authors agree there are no conflicts to declare.

## Supplementary Material

RA-009-C9RA07630A-s001
