# Supplementary material for: Green synthesis of zinc oxide nanostructures and investigation of their photocatalytic and bactericidal applications
Source: RSC Adv. 2019 Nov 18;9(63):36967–81. doi: 10.1039/c9ra07630a (PMC9075592; doi:10.1039/c9ra07630a)
Supplement: RA-009-C9RA07630A-s001 [file RA-009-C9RA07630A-s001.pdf]

Electronic Supplementary Material (ESI) for RSC Advances.
This journal is © The Royal Society of Chemistry 2019

Synthesis of Zinc Oxide Nanostructures and Investigation of their Photocatalytic and Bactericidal Applications

Mebrahtu Hagos Kahsay *[a], Aschalew Tadessb [b], Dharamasoth RamaDevi [c], Neway Belachew [d], and K. Basavaiah [e]

[a]*Department of Chemistry, Woldia University, Woldia,* P.O.BOX *400, Ethiopia.*

[b]*Department of Applied Chemistry, Adama Science and Technology University, Adama,* P.O.BOX *1888, Ethiopia.*

[c]*AU College of Pharmaceutical Sciences, Andhra University, Visakhapatnam, 530003, India.*

[d]*Department of Chemistry, Debre Berhan University, Debre Berhan,* P.O.BOX *445, Ethiopia.*

[e]*Department of Inorganic & Analytical Chemistry, Andhra University, Visakhapatnam, 530003, India.*

[*]**Corresponding author**.

E-mail address: hagosmebrahtu@gmail.com (**Kahsay, Mebrahtu Hagos**)

LIST OF TABLES

Table 1 Preliminary phytochemical investigation of aqueous leaf extract of *Dolichos lablab* L.

S. No.	Test	Chemical Constituents	Result
1	Mayer's test	Alkaloid	+
2	$FeCl_3$ solution	Phenol	+
3	NaOH	Flavonoid	+
4	Fehling solution	Carbohydrate	–
5	Conc. HNO_3	Amino acid and protein	+
6	Methanol, chloroform, H_2SO_4	Terpenoid	+
7	Shake with water	Saponin	+

Where, (+) indicates presence and (-) indicates absence

Table 2 Structure and geometric parameters of ZnO nanostructures

2θ (degree)	FWHM (β)	d-spacing (Å)	Cos θ	Crystallite size (nm)
8.3932	1.3056	10.52625	0.99732	6.38
31.8365	0.4080	2.80859	0.96165	21.16
34.5150	0.2448	2.59651	0.95498	35.51
36.3340	0.1224	2.47059	0.95015	71.38
47.6293	0.5712	1.90772	0.91486	15.88
56.6423	0.0816	1.62369	0.88030	115.55
62.9124	0.2448	1.47610	0.85304	39.75
66.5366	0.5712	1.40422	0.83611	17.38
68.0506	0.4488	1.37662	0.82879	22.31
69.1192	0.6528	1.35792	0.82354	15.44
**Average**				**36.07**

Table 3 Zone of inhibition of ZnO nanostructures against pathogens

S. No.	Pathogens	Concentration of ZnO nanostructures (mg/ml)			Standard (Ciprofloxacin)	Control (DMSO)
		2.5	5.0	10.0	5 µg/ml	
1	*Bacillus pumilis*	12 mm	18 mm	15 mm	40 mm	NA
2	*Sphingonomas paucimobilis*	15 mm	20 mm	15 mm	40 mm	NA

Where, DMSO is Dimethyl sulfoxide and NA is Not available
